# Supplementary material for: Femtosecond laser writing of ant-inspired reconfigurable microbot collectives
Source: Nat Commun. 2024 Aug 23;15:7253. doi: 10.1038/s41467-024-51567-4 (PMC11343760; doi:10.1038/s41467-024-51567-4)
Supplement: Supplementary file 1 — Supplementary Information [file 41467_2024_51567_MOESM1_ESM.pdf]

Supplementary Information

Femtosecond laser writing of ant-inspired reconfigurable microbot collectives

Zhongguo Ren[1], Chen Xin[1,2*], Kaiwen Liang[1], Heming Wang[1], Dawei Wang[1], Liqun Xu[1], Yanlei Hu[1], Jiawen Li[1], Jiaru Chu[1], and Dong Wu[1*]

[1]Key Laboratory of Precision Scientific Instrumentation of Anhui Higher Education Institutes, CAS Key Laboratory of Mechanical Behavior and Design of Materials, Department of Precision Machinery and Precision Instrumentation, University of Science and Technology of China, Hefei 230026, China.

[2]Department of Mechanical and Automation Engineering, The Chinese University of Hong Kong, Hong Kong 999077, China

E-mail: xinc@ustc.edu.cn and dongwu@ustc.edu.cn

This PDF file includes:

Supplementary Figure 1 to Figure 22

Supplementary Note 1

Supplementary Figures

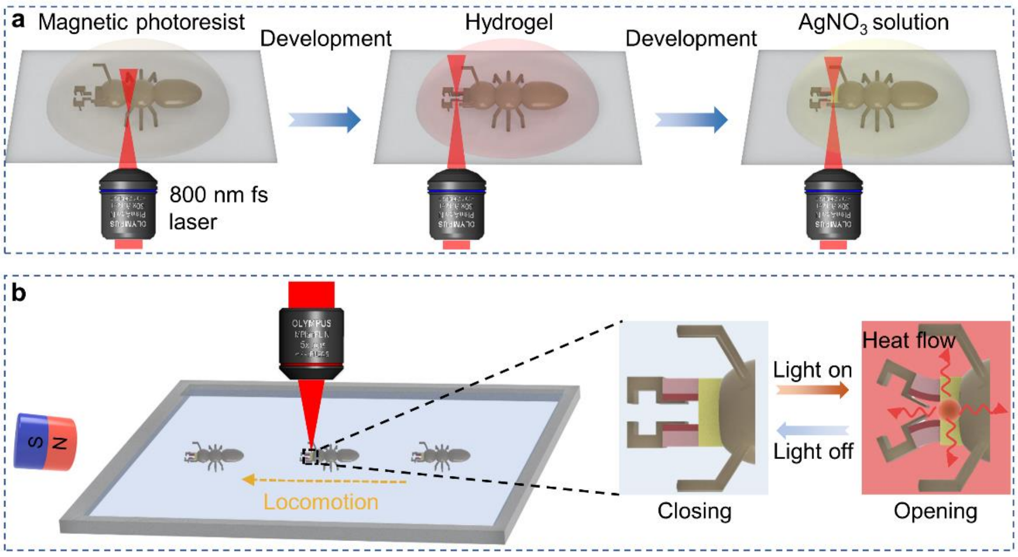

Supplementary Figure 1. Schematics of multi-step two-photon polymerization (TPP) process and assembly. (**a**) The whole procedure involves three steps. The magnetic photoresist body and mandibles are printed first, then the asymmetric hydrogel joints are printed to connect the body and mandibles. Finally Ag NPs are reduced to the head based on photoreduction. Development is required between each of these steps. (**b**) Our assembly is realized under the coordinated control of magnetic and light fields, where the magnetic field controls the ant microbot's motion and the light field controls the switching of the ant microbot's mandibles. The wavelength of the laser used to fabricate and actuate the ant microbot is 800 nm.

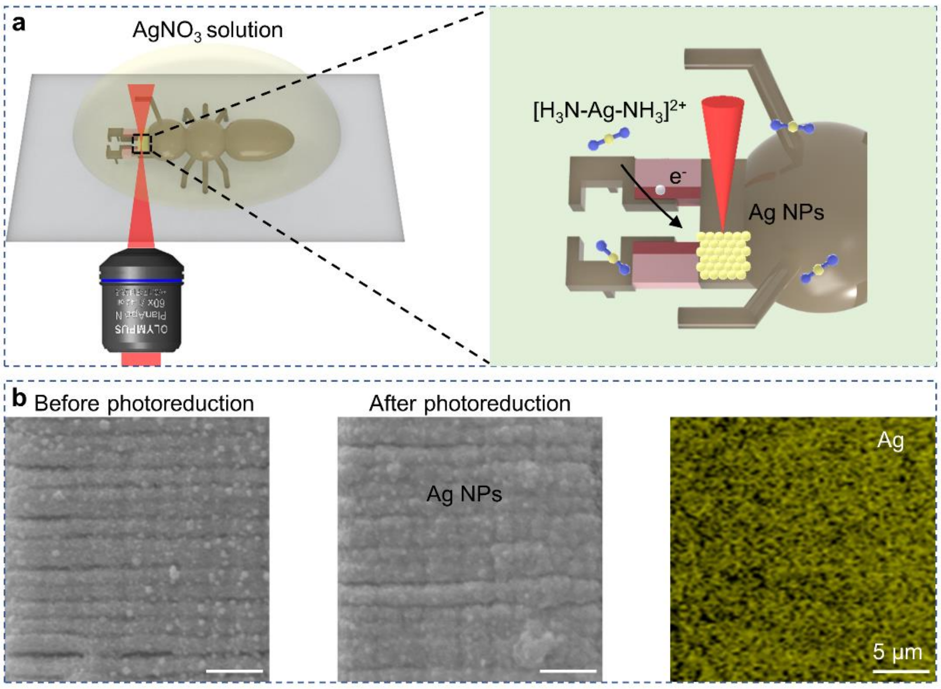

Supplementary Figure 2. (**a**) The schematic shows the process of silver nanoparticles (Ag NPs) photoreduction. Under femtosecond laser irradiation, reduction occurs immediately due to the two-photo absorption, which induces the silver ammonium ions to be reduced to Ag NPs. (**b**) The SEM images present the state before and after the Ag NPs photoreduction, and the EDS image also confirms the presence of the Ag NPs.

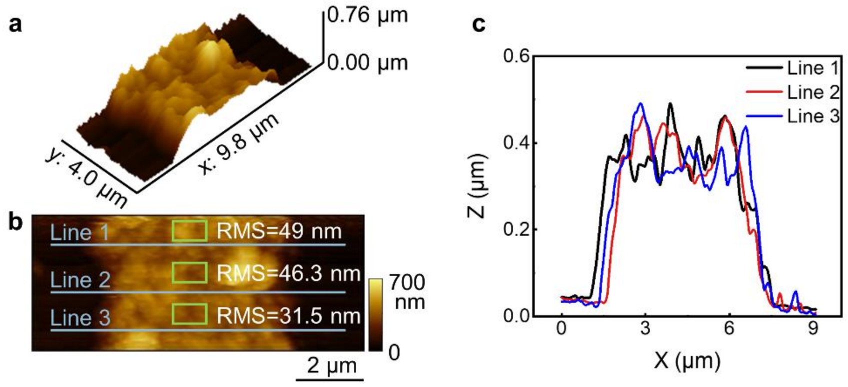

Supplementary Figure 3. Characterization of thickness and roughness of Ag NPs layer at a laser power of 3 mW. (**a**) 3D image of Ag NPs layer scanned by AFM. (**b**) The roughness of the three regions of the Ag NPs layer is 49 nm, 46.3 nm, and 31.5 nm, respectively. RMS indicates the root mean square value of roughness. (**c**) From the thickness of the Ag NPs layer on the random extraction lines, it can be concluded that the thickness of the entire Ag NPs layer is ~350 nm.

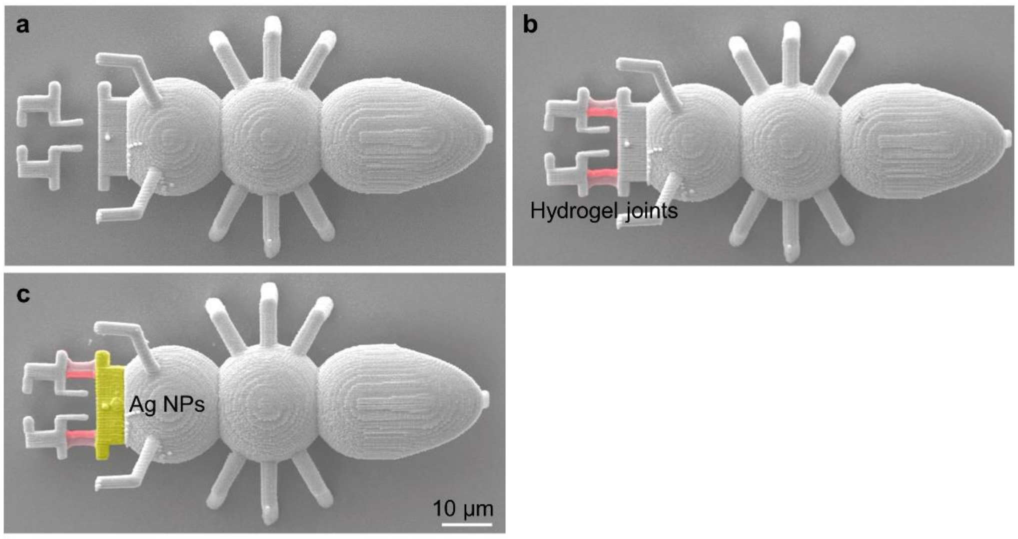

Supplementary Figure 4. The SEM images of each processing step. (**a**) The SEM image shows the magnetic photoresist ant microbot body and mandibles after the first step of the TPP process. (**b**) The SEM image shows the integration of hydrogel joints into the magnetic photoresist body after the second step of the TPP process. (**c**) The SEM image shows the complete ant microbot after photoreduction.

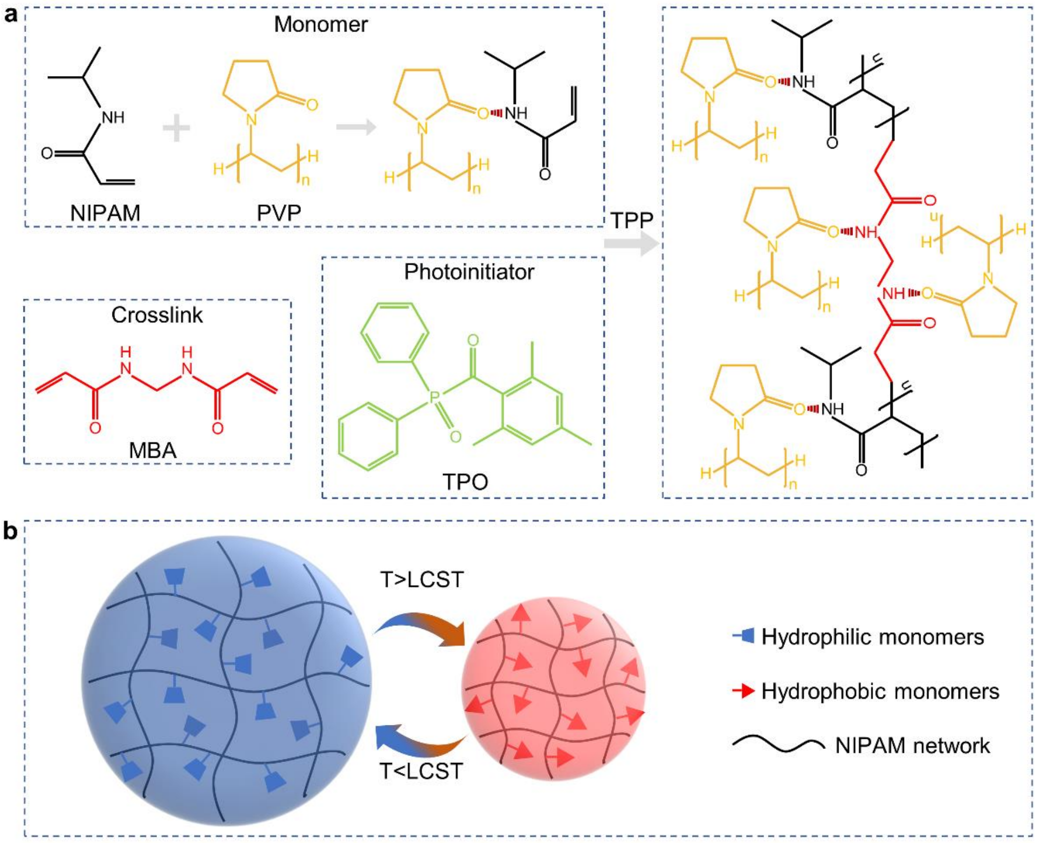

Supplementary Figure 5. Composition and deformation mechanism of the thermal stimuli-responsive hydrogel. (**a**) The components of this hydrogel include monomer (N-Isopropyl acylamide, NIPAM), crosslinker (Methylene-Bis-Acrylamide, MBA), photoinitiator (Diphenyl (2,4,6-tri-methyl benzoyl) phosphine oxide, TPO), and polyvinylpyrrolidone (PVP). When the hydrogel is polymerized, hydrogen bonding occurs between NIPAM and PVP to enhance the mechanical properties of the hydrogel. (**b**) The deformation mechanism of thermo-responsive hydrogel is thought to be a reversible phase transition from a contracted hydrophobic state at temperatures above its lower critical solution temperature (LCST) to a hydrophilic swollen state at temperatures below its LCST, which is almost between 32°C and 33°C.

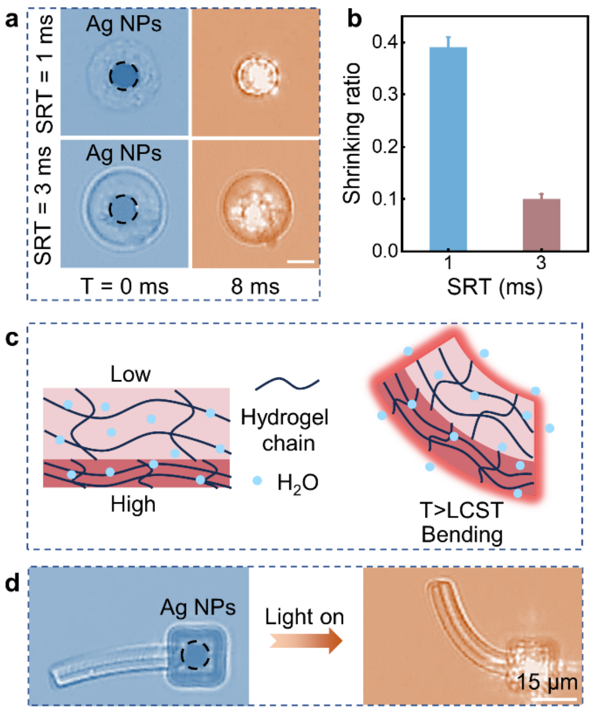

Supplementary Figure 6. (**a**) The microscope images show the shrinkage of hydrogel microplates with scanning repeat time (SRT) of 1 ms and 3 ms, respectively. (**b**) Quantitative statistics of shrinkage ratio of hydrogel microplates with SRT of 1 ms and 3 ms. The error bars represent the standard error of the measurements. (n = 3) (**c**) The schematic of joint with different SRT. In order to realize the bending deformation of the joint, it is designed so that the width ratio of the SRT = 1 ms region to the total width is 0.7 and the remaining part of SRT = 3 ms. When the joint's temperature is higher than the LCST of the hydrogel via surface incorporated photo absorbers, it immediately bends toward the side of the fewer SRT due to the larger shrinking ratio of the fewer SRT than the more SRT. (**d**) The microscope images illustrate the corresponding initial and bending states of the joint.

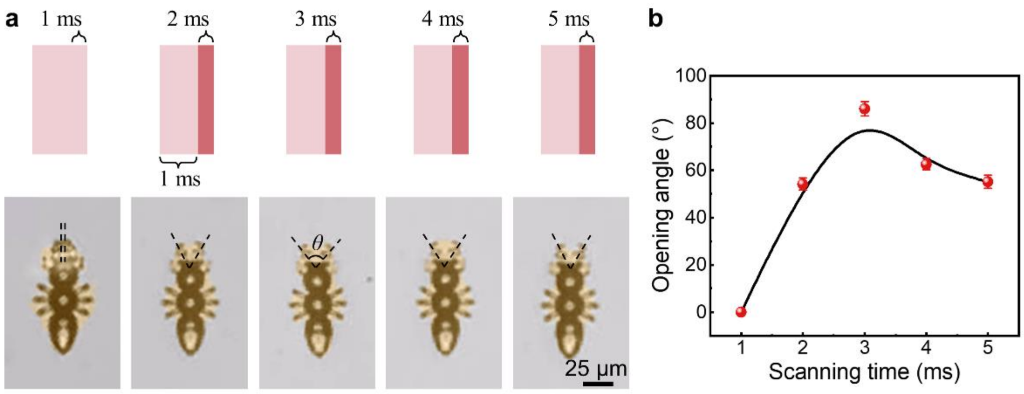

Supplementary Figure 7. Effect of the scanning time of the high crosslink density layer in the hydrogel joint on the mandible opening angle. (**a**) The opening angle increases and then decreases with increasing scanning time of the high crosslink density layer. (**b**) Quantitative statistics of the opening angle and the scanning time of the high crosslink density layer. The error bars represent the standard error of the measurements (*n* = 3).

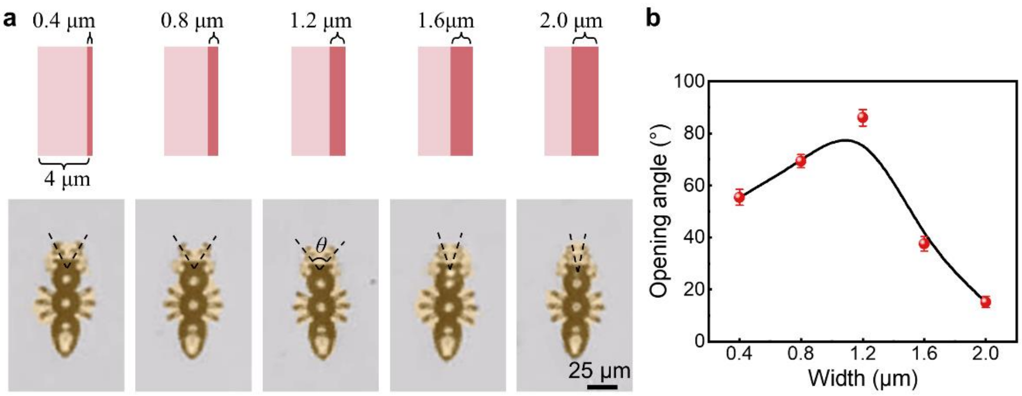

Supplementary Figure 8. Effect of the width of the high crosslink density layer in the hydrogel joint on the mandible opening angle. (**a**) The opening angle increases and then decreases with the increasing the width of high crosslink density layer. (**b**) Quantitative statistics of the opening angle and the width of high crosslink density layer. The error bars represent the standard error of the measurements (n = 3).

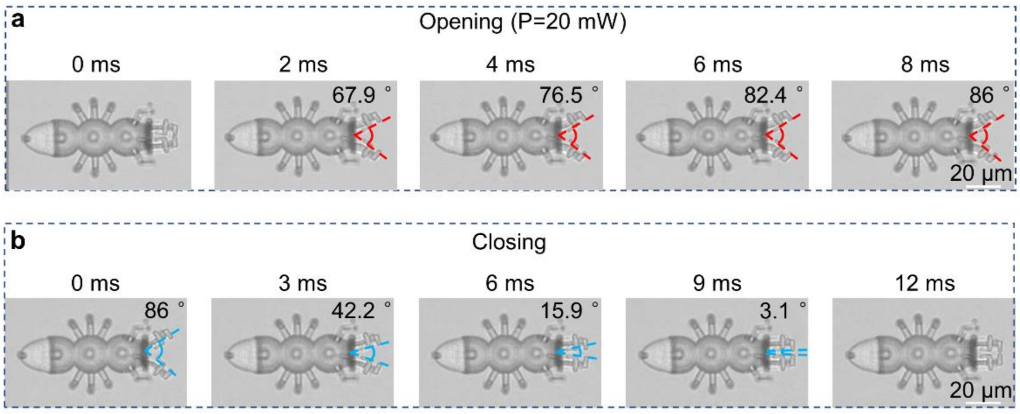

Supplementary Figure 9. (**a-b**) Time-lapse images of ant microbot mandibles opening with light on and closing with light off at a high-speed camera (1000 fps), respectively. At a laser power of 20 mW, the ant microbot mandibles are able to completely open to the maximum value of 86° within 8 ms, and when the light is turned off, the ant microbot mandibles take 12 ms to fully close. This is because that the heating process of the hydrogel joints, which benefits from high energy density, is faster than the cooling process.

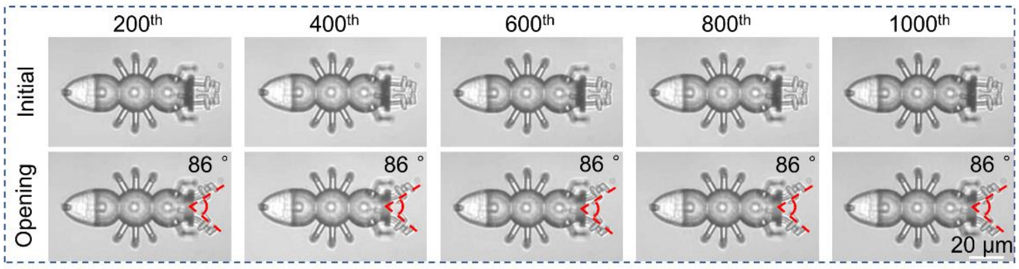

Supplementary Figure 10. (**a**) The microscope images show the initial and the opening state of the ant microbot mandibles at the 200th, 400th, 600th, 800th, and 1000th cycle, respectively, and the results demonstrate that the ant microbot mandibles have a promising deformation stability.

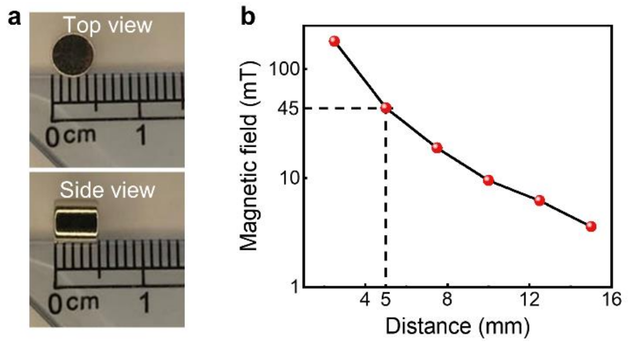

Supplementary Figure 11. (**a**) A cylindrical permanent magnet with a diameter and height of 5 mm is used to manipulate the movement of the ant microbot. (**b**) The relationship between the magnetic field strength and the distance. In our experiments, the distance from the magnet to the ant microbot is about 5 mm, that is, the strength of the applied magnetic field is about 45 mT.

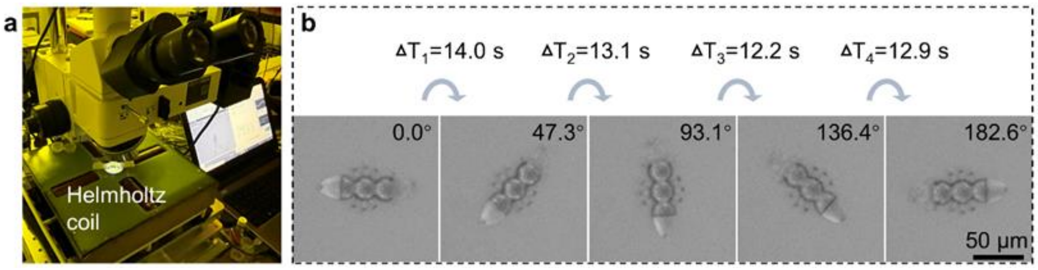

Supplementary Figure 12. Rotational motion testing. The test is performed by generating a uniform magnetic field in a horizontal plane through the Helmholtz coil. Initially, the ant microbot's head is oriented toward the positive direction of the X-axis, and when a uniform magnetic field (~45 mT) is applied by the Helmholtz coil along 45° counterclockwise, the ant microbot would rotate counterclockwise following the direction of the magnetic field. Repeating the above steps four times. (**a**) Setup of the rotational motion testing. (**b**) The time-sequence optical images of rotational motion.

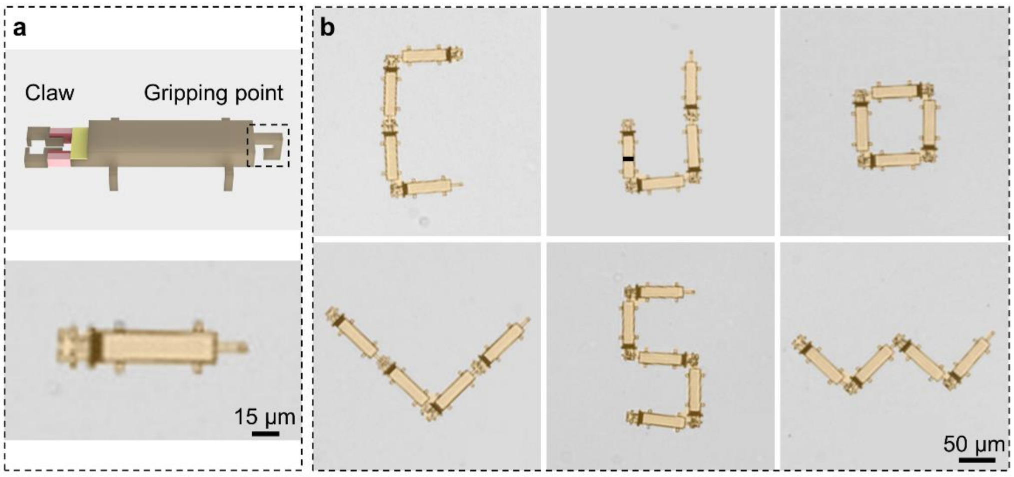

Supplementary Figure 13. Simplified rectangular-shaped microbot. (**a**) Schematic diagram and optical image of a rectangular-shaped microbot. (**b**) Optical images of C, J, O, V, S, and W shape assembled from rectangular-shaped microbots.

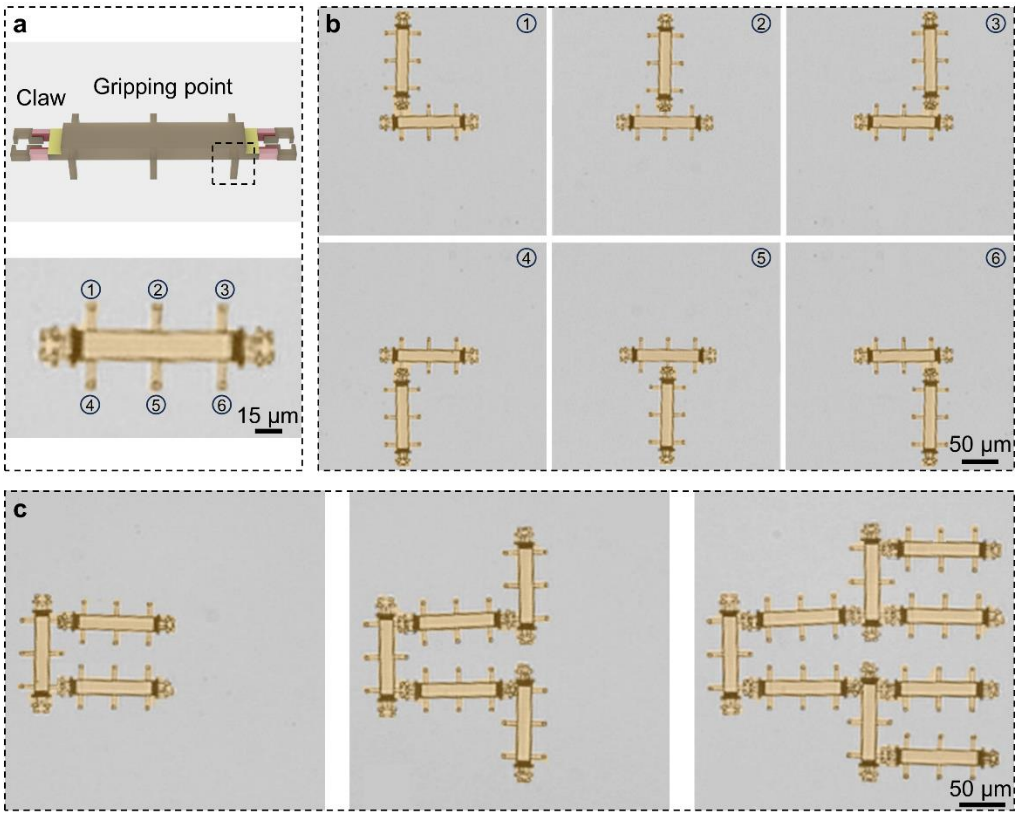

Supplementary Figure 14. A rectangular-shaped microbot has two claws and six legs on its body, and each leg can be used as a gripping point. (**a**) Schematic diagram and optical image of the microbot. (**b**) Optical images of each leg of the microbot used as a gripping point for assembly. (**c**) Optical diagrams of complex bifurcated tree-like patterns assembled from the microbots.

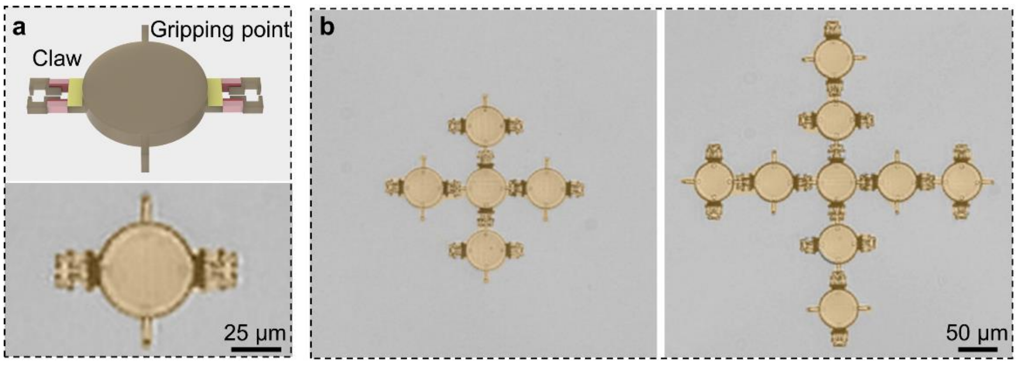

Supplementary Figure 15. A disk-shaped microbot with two claws and two gripping points. (**a**) Schematic diagram and optical image of a disk-shaped microbot. (**b**) Optical diagrams of shapes dispersed from the center outward assembled from different numbers of disk-shaped microbots.

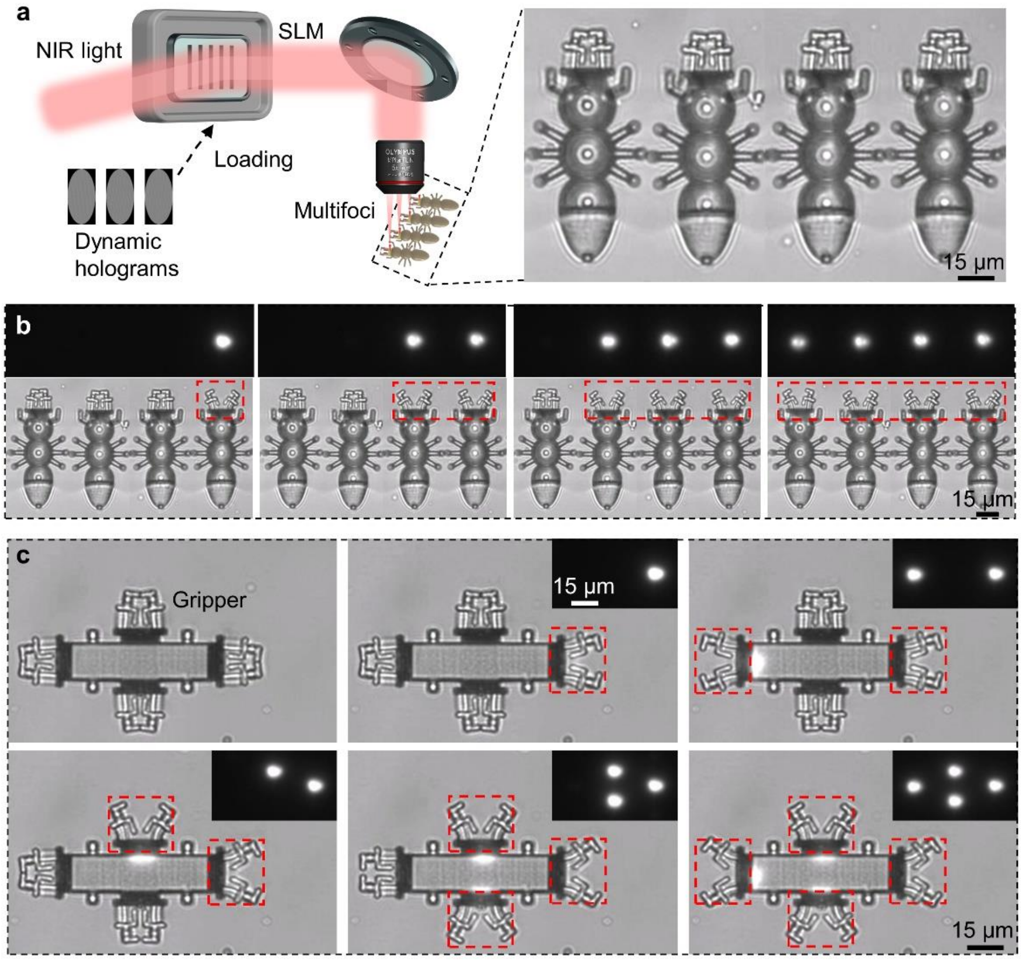

Supplementary Figure 16. A SLM is utilized to modulate multiple beams of light to actuate several grippers open. (**a**) Schematic diagram of the multifocal light field actuation. (**b**) Optical diagram of multifocal light fields driving the opening of multiple ant microbot grippers. (**c**) Optical diagram of multifocal light fields driving multiple grippers opening of a single microbot.

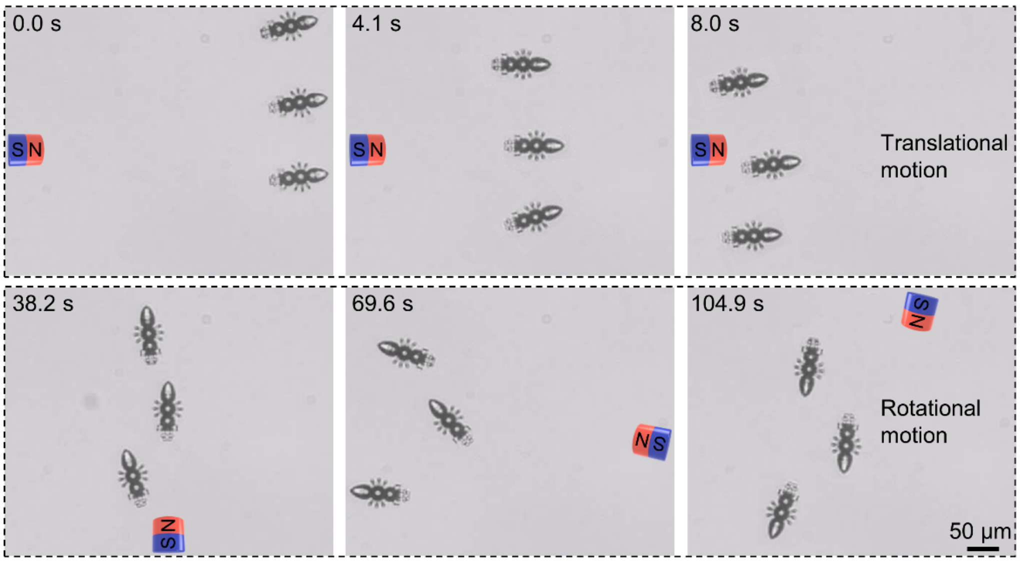

Supplementary Figure 17. The time-sequence optical images of synchronized motion of ant microbots prepared from the same magnetic material driven by the same magnetic field.

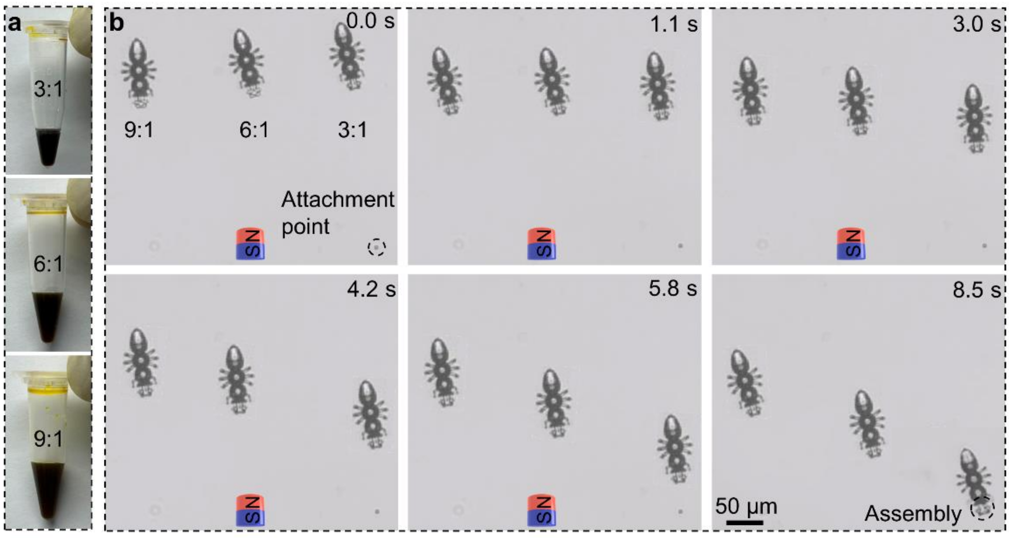

Supplementary Figure 18. Movement of ant microbots prepared from different magnetic materials driven by the same magnetic field. (**a**) Different magnetic materials prepared. (**b**) The time-sequence optical images of motion of ant microbots prepared from different magnetic materials driven by the same magnetic field.

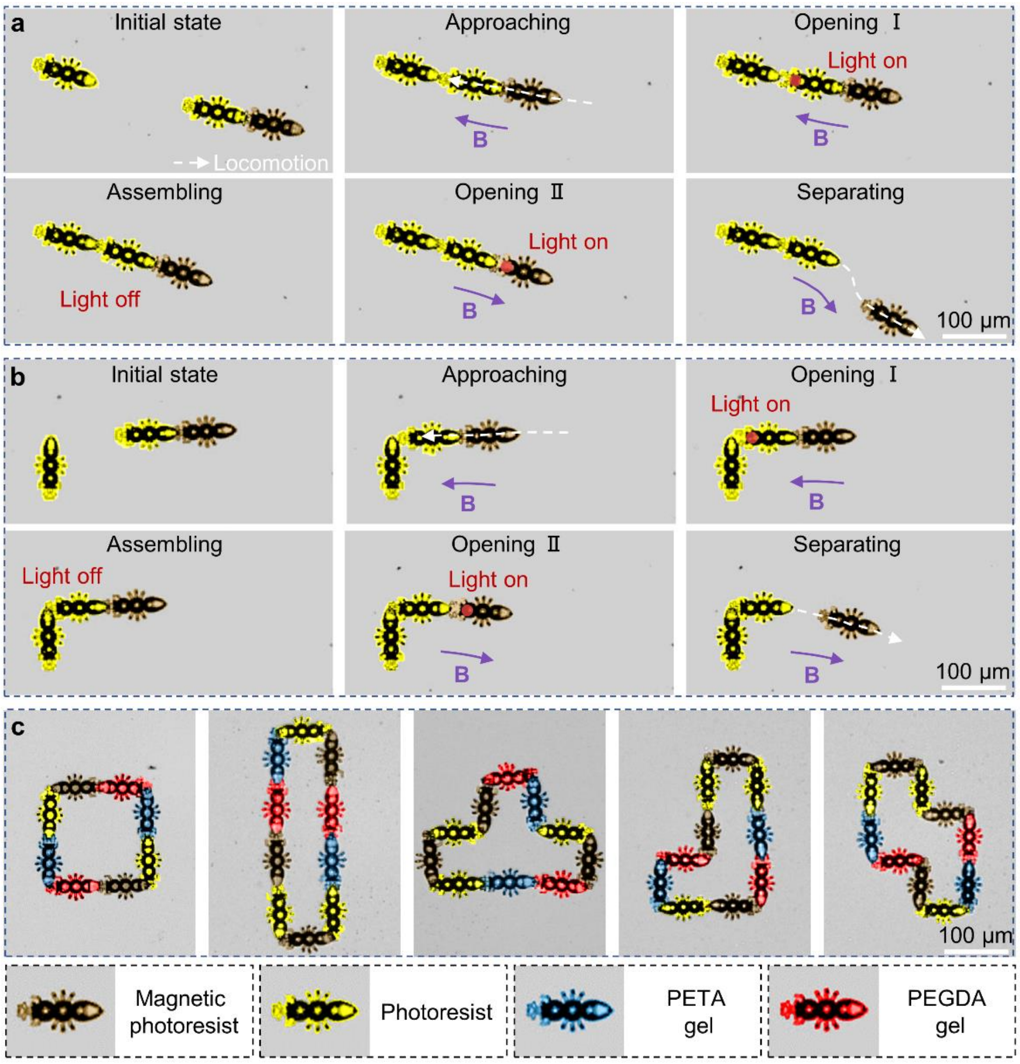

Supplementary Figure 19. (**a**-**b**) The time-sequence optical images show two non-magnetic ant microbots guided by a magnetic ant microbot to achieve 180° and 90° assemblies through the synergistic control of magnetic and light fields, respectively. (**c**) Multi-material ant microbots are patterned into various configurations that have close resemblance to the tetrominoes in the popular game Tetris by way of the proposed magnetic ant microbot-assisted assembly strategy, where orange indicates magnetic photoresist, yellow indicates traditional photoresist SZ2080 (zirconium/silicon hybrid sol-gel), blue indicates PETA (pentaerythritol triacrylate) gel, and green indicates PEGDA (poly (ethylene glycol) diacrylate) gel.

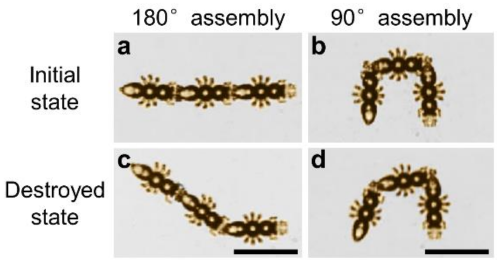

Supplementary Figure 20. Optical comparison images between before and after the assembled microstructures are destroyed. (**a**-**b**) Optical images of 180° assembly and 90° assembly before destruction. (**c**-**d**) Optical images of 180° assembly and 90° assembly after destruction. Scale bar, 100 μm.

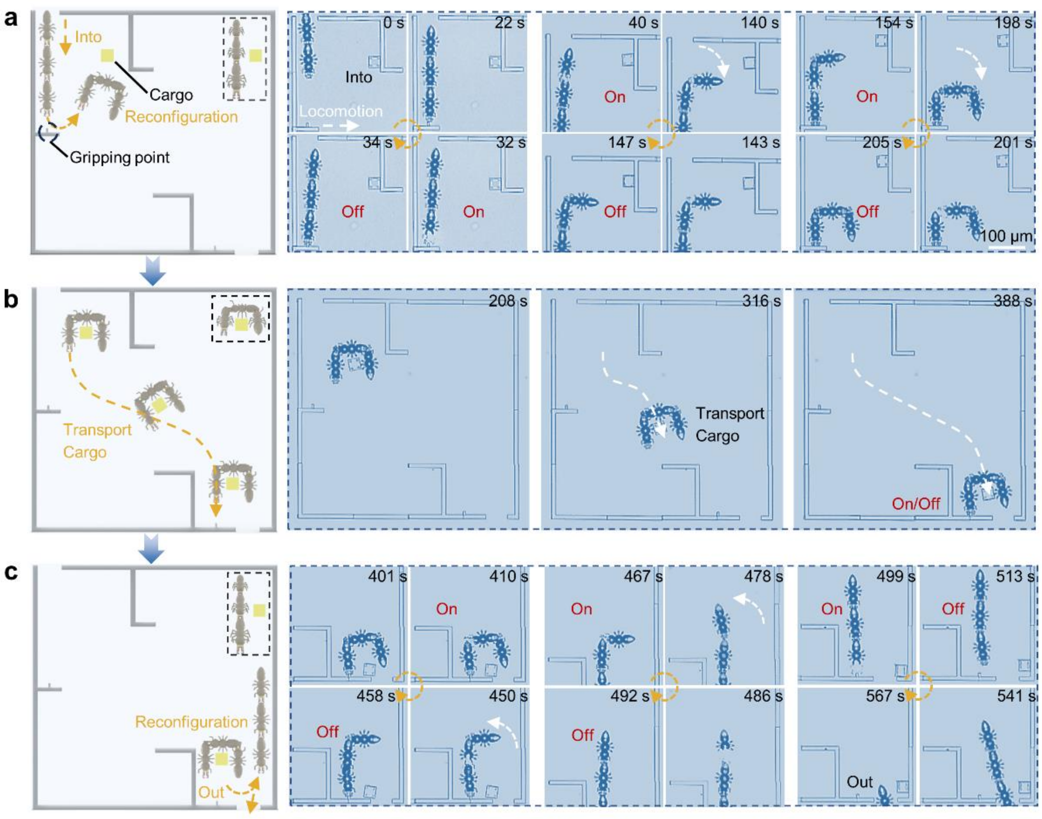

Supplementary Figure 21. Maze navigation and cargo delivery. (**a**) The connected three ant microbots are initially located outside the entrance of the maze and are assembled in a 180° method so that they can be pulled through a narrow channel at the entrance using a magnetic field. Once inside the maze, the whole structure is temporarily fixed by the mandibles of the first unit grasping the contact point, and the purpose of clamping is to selectively manipulate one unit without affecting neighboring units to adjust the assembly pattern of the three units. Then, again under the coordination control of the magnetic and optical fields, the three assembled units are transformed from I shape to L shape and then to C shape to be able to carry a cargo. The cargo is a cubic photoresist block (20 μm in length) processed by TPP. We conduct this segment three times. (**b**) With the cargo in their grasp, the C-shaped three-unit microbot is transported magnetically to the exit of the maze. We conduct this segment one time. (**c**) The C-shaped three-unit microbot is reconfigured to an L shape and then to the initial I shape under the coordinated control of the magnetic and optical fields in order to release the cargo as well as to be able to exit the maze. Finally, the three-unit microbot assembled in the I shape is successfully disengaged from the contact point and maneuvers out of the maze under the manipulation of the magnetic and light fields. We conduct this segment three times.

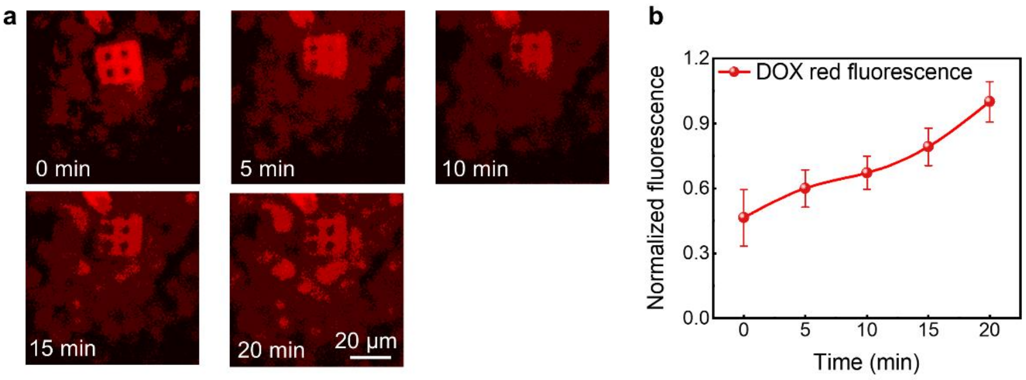

Supplementary Figure 22. (**a**) Red fluorescence comparison images show the red fluorescence changes of HeLa cells cultured in square micro-fences with a doxorubicin hydrogel block inside in a time span of 20 min. (**b**) Quantitative statistics of red fluorescence intensity of HeLa cells with a doxorubicin hydrogel block inside, demonstrating that drug could diffuse efficiently from the doxorubicin hydrogel block. The error bars represent the standard error of the measurements (n = 3).

Supplementary Notes

Supplementary Note 1. Simulation of the joint deformation.

The numerical simulations are conducted in the commercial finite-element software COMSOL, and the Solid Mechanics module in COMSOL is accounted for the thermomechanical deformation calculations of the ant microbot. For the bending analysis, the degree of deformation of the structures with different crosslink densities to temperature changes is introduced as a thermal strain. We choose the experimental results of shrinkage of two hydrogel microplates with different SRT as the numerical results in the simulation model. In this way, we obtain thermal shrinkage ratio $\alpha_1 = 0.39$ and $\alpha_2 = 0.1$ for the hydrogel microplates with SRT of 1 ms and SRT of 3 ms, respectively. For the heat diffusion calculations, we construct the ant microbot model in a sufficiently large water bath and set the outer boundaries of the water bath to room temperature while applying a Gaussian-distributed incident laser beam to the surface of the ant microbot head.
