# Supplementary material for: Femtosecond laser writing of ant-inspired reconfigurable microbot collectives
Source: Nat Commun. 2024 Aug 23;15:7253. doi: 10.1038/s41467-024-51567-4 (PMC11343760; doi:10.1038/s41467-024-51567-4)
Supplement: Supplementary file 3 — Description Of Additional Supplementary File [file 41467_2024_51567_MOESM3_ESM.pdf]

Description of Additional supplementary files

Supplementary Movie 1. Light-triggered micromandibles with short response time (~10 ms).

Supplementary Movie 2. Light-triggered opening of micromandibles (1000 times).

Supplementary Movie 3. Magnetic and light driven 180° assembly and separation of ant microbot.

Supplementary Movie 4. Magnetic and light driven 90° assembly and separation of ant microbot.

Supplementary Movie 5. Magnetic unit guides the 180° assembly of non-magnetic units.

Supplementary Movie 6. Magnetic unit guides the 90° assembly of non-magnetic units.

Supplementary Movie 7. Robustness testing of the 180° assembly.

Supplementary Movie 8. Robustness testing of the 90° assembly.

Supplementary Movie 9. Gap traversal ability of the assembled unit.

Supplementary Movie 10. Maze navigation and cargo transport.
